# Injuries sustained by earthquake relief workers: a retrospective analysis of 207 relief workers during Nepal earthquake

**DOI:** 10.1186/s13049-016-0286-4

**Published:** 2016-07-26

**Authors:** Feizhou Du, Jialing Wu, Jin Fan, Rui Jiang, Ming Gu, Xiaowu He, Zhiming Wang, Ci He

**Affiliations:** 1 Department of Radiology, Chengdu Military General Hospital, NO.270, Rongdu Avenue, Jinniu District, Chengdu, Sichuan 610083 China; 2 Department of Neurology, Chengdu Military General Hospital, Chengdu, 610083 China; 3 Department of Radiology, No. 8 Hospital of PLA, Shigatse, Tibet 857000 China; 4 Department of Radiology, No. 41 Hospital of PLA, Shannan, Tibet 856100 China

**Keywords:** China, Earthquake, Fracture, Tibet plateau, Trauma, Rescue

## Abstract

**Background:**

This study aimed to analyse the injuries sustained by rescue workers in earthquake relief efforts in high altitude areas for improving the ways of how to effectively prevent the injuries.

**Methods:**

The clinical data of 207 relief workers from four military hospitals in Tibet, who were injured in the Tibetan disaster areas of China during ‘4.25’ Nepal earthquake rescue period, was retrospectively analyzed. The demographic features, sites of injury and causes of injury were investigated.

**Results:**

The most frequently injured sites were the ankle-foot and hand-wrist (*n* = 61, 26.5 %), followed by injuries in leg-knee-calf (*n* = 22, 9.6 %), head-neck (4.87 %), thoracic and abdominal region (2.6 %) and lower back (3.9 %). The specific high-altitude environment increased the challenges associated with earthquake relief.

**Discussion:**

The specific plateau environment and climate increased the burden and challenge in earthquake relief. The injury distribution data shown in this study demonstrated that effective organization and personnel protection can reduce the injury occurrences.

**Conclusion:**

Relief workers were prone to suffering various injuries and diseases under specific high-altitude environment.

## Background

On 25 April 2015 at 14:11, local time, a 7.8-magnitude (Mw) earthquake occurred in Nepal (‘4.25’ Nepal Earthquake). The impact of the earthquake, which was followed by hundreds of aftershocks, has led to at least 8,000 deaths and more injuries. Additional casualties were reported in Chinese Tibet, India, Bangladesh and Bhutan. On 15^th^ May 2015, Sonam Dhargay of the China Network (http://sc.cnr.cn/sc/2014sc/20150427/t20150427_518404301.shtml) reported from Lhasa that, “By May 25^th^, 2015, 27 deaths were caused by the earthquake in Shigatse, three people were missing, 860 people were injured, and 300,507 people from 41,677 households were affected. The Tibetan government has implemented a rescue force of 843,096 shifts, 39,073 vehicles and all types of equipments”.

The city of Shigatse, 43 km from the epicentre in Chinese Tibet, was damaged by the earthquake with serious loss of life and property. Shigatse is located in the southwestern Qinghai-Tibet plateau of China at an average elevation of more than 4000 m (high altitude) with a large diurnal temperature variation, less oxygen levels and strong ultraviolet radiation in the daytime [1, 2]. Its specific climate and geographical environment present great challenges to earthquake relief work [1, 2]. Almost at the same time of earthquake, Tibetan medical institutions at all levels rushed emergency medical teams to the earthquake relief front where several field ambulatory hospitals were constructed to provide first line of medical support. From the patient records at these medical institutions, the disaster relief workers were accounted for a large proportion.

Most of the published studies were focused on the injuries of all the earthquake victims rather than relief personnel themselves [3–6]. In specific geographical areas and climates, such as the Tibet plateau, injuries and diseases of relief workers are more frequent and could become a burden on local authorities [2, 7]. This study aimed to retrospectively analyse the injuries sustained by rescue workers in the earthquake relief efforts for improving the ways of how to effectively prevent the injuries. Therefore, the clinical data of in-patient and out-patient injured relief workers from four military hospitals in Tibet were collected and analysed. Additionally, the characteristics and causes of the injuries were investigated.

## Methods

### Data collection

Written informed consent was waived for secondary use of health data. Out-patient and in-patient records were collected from General Hospital of Tibetan Military Region, No. 8 Hospital of the People’s Liberation Army (PLA), No. 115 Hospital of PLA and No. 41 Hospital of PLA during the 20 days period after the earthquake. Patients’ records were collected when they met the criteria as follows: (1) the causes of injury or illness were related to participation in Shigatse disaster relief following the ‘4.25’ Nepal earthquake; (2) injuries caused by aftershocks during the process of disaster relief; (3) diagnosis based on clinical features, laboratory examination and/or digital radiography (DR) or multi-layer spiral computed tomography (CT) findings and (4) no history of surgery or treatment prior to visiting the clinic. To avoid bias, we eliminated patients whose conditions were related to motor vehicle accidents during disaster relief efforts. This study used descriptive statistical methods to analyse the population structure, types of injury, injury positions and relevant pathogenic factors. Initial screening generated 239 cases, from which 32 cases were excluded due to failure to meet aforementioned criteria. The remaining 207 cases were included for further analyses. This study was approved by the Medical Ethics Committee of Chengdu Military General Hospital.

### Research variables

Original patients’ records included 23 variables, after reviewing clinical and laboratory data by two clinical experts and imaging data by two imaging experts; ten variables were included for further analyses. The ten included variables are sex, age, occupation, injury type, injury position, cause of injury, medical treatment, laboratory findings, physical examinations, and DR or CT. Based on the International Classification of Diseases (ICD)-10 (version 2014) [8], standards were used to record injuries at the institutes according to the anatomical parts of the body; i.e. a fracture and adjacent soft tissue injury at the same anatomical site were classified as a single position injury, whereas injuries at different anatomical sites in the same patient were classified as a multiple position injury. Occupational classifications included soldiers, farmers and herdsmen, engineers, firemen, medical staff and others, these classifications were further grouped into three relief working groups according to their personal protective equipment (PPE) during the relief working process: Military (soldiers); Professional (engineers, medical staff and firemen); Others (farmers, herdsmen and others). For analysis, the causes of injuries were divided into two major categories, traumatic injuries and non-traumatic injuries. The former included injuries from a falling object or crush injuries, falls and sprains, whereas the latter included factors related to the climate, environmental exposure and diet. The hypothesis in the present study was that the injuries occurred for professional rescue workers in the earthquake rescue work might have some common characteristics, which are useful to prevent the injuries in the future rescue work.

### Statistical analysis

Double data entry was used to minimize data entry errors. SPSS16.0 (SPSS Inc, Chicago, IL, USA) statistical package was used to conduct statistical analysis. Continuous data was expressed as mean ± standard deviation and Pearson chi-square test was used for categorical data. One-way ANOVA was used to determine the significance of the difference among the group means. An alpha level of *P* < 0.05 was used to determine the significance.

## Results

### Patient demographic and clinical characteristics

**Fig. 1:**
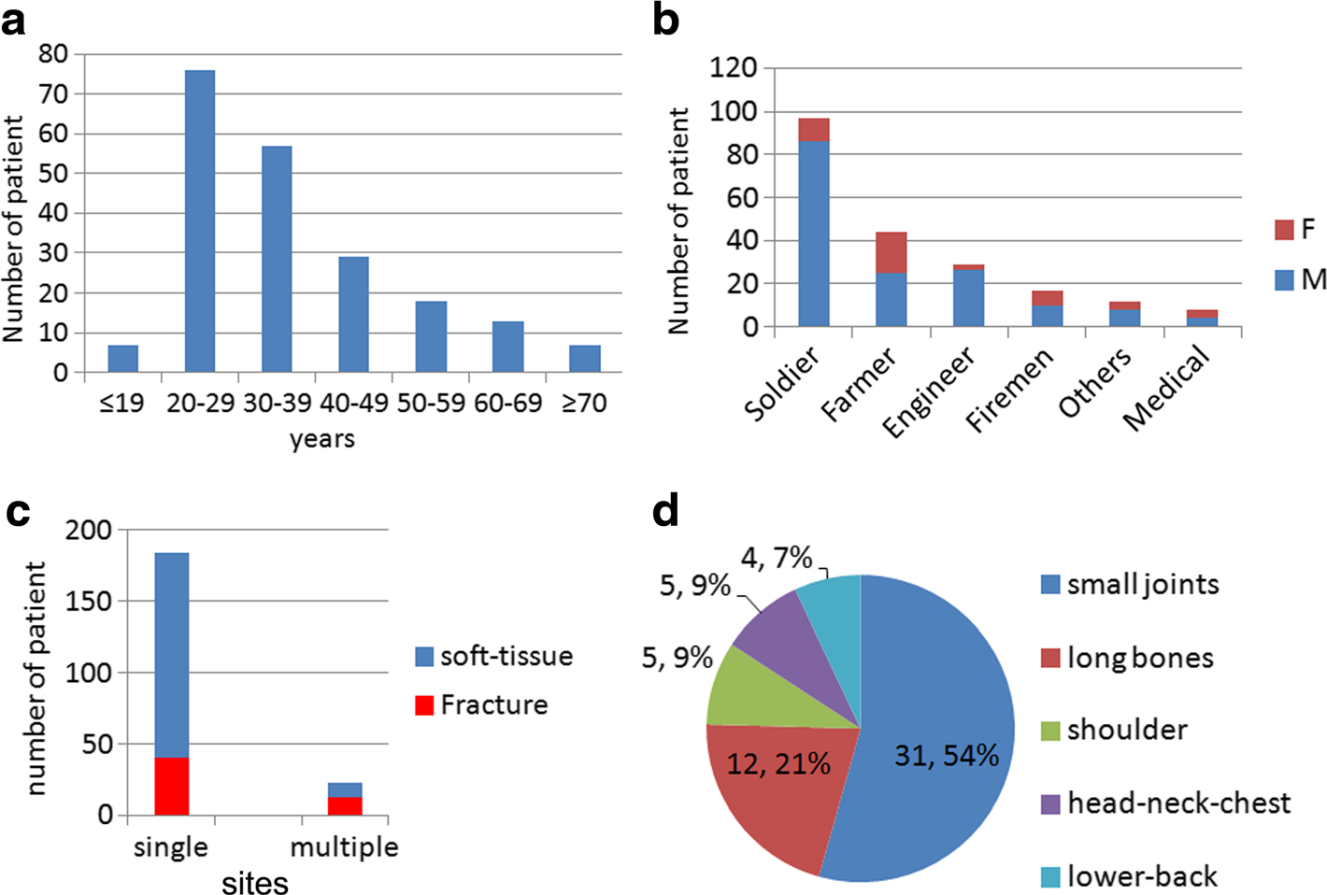
Patient demographic and clinical characterics. **a** age distribution of patients **b** occupation and sex distribution of patients **c** injury sites of traumatic injuries **d** anatomical sites of fracture injuries

All of the 207 patients were disaster relief rescuers, most of them were young males. The mean age of these patients was 36.15 ± 13.72 years. The age distribution heavily skewed to 20-40 years old, with a total of 133 patients (approximately 64.3 %) aged 20–39 years (Fig. 1a, Table 1). A significantly higher proportion was male (159, 76.8 % male vs. 48, 23.2 % female patients, Table 1); additionally, all of the 23 patients with multiple-site-injuries were male. Most of the patients were rescuers from military working group, which accounted for 97 cases (46.8 %). A detailed distribution of patients’ professions was shown in Fig. 1b.

**Table 1:** Demographic feature of 207 rescuer patients

		Sex		
		Male(*n* = 159)	Female(*n* = 48)	Total(*n* = 207)
Age(year)	N(%)			
	≤19	6 (2.9)	1 (0.5)	7 (3.4)
	20–29	62(30.0)	14(6.8)	76(36.7)
	30–39	42(20.3)	15(7.2)	57(27.5)
	40–49	19(9.1)	10(4.8)	29(14.0)
	50–59	12(5.8)	6 (2.9)	18(8.7)
	60–69	12(5.8)	1 (0.5)	13(6.3)
	≥70	6 (2.9)	1 (0.5)	7 (3.4)
Relief working group,	N(%)			
	Military	86(41.5)	11(5.3)	97(46.8)
	Professional	40 (19.3)	14 (6.7)	54 (26)
	Others	33 (16)	23 (11.1)	56(27.1)

The evaluation of injury sites (Fig. 1c) showed 184 cases (88.9 %) with single-site-injuries, a significantly higher number than that of multiple-site-trauma cases (23 cases, 11.1 %). The 23 multiple–site-trauma cases were all double site injuries and no patients with three or more injured areas were observed. Among all 207 patients, 115 cases experienced traumatic injuries and 92 cases experienced non-traumatic injuries. A total of 138 traumatic injury sites were further classified into two categories and was shown in Table 2: soft tissue injuries (81, 35.2 %) or fractures (57, 24.8 %).

**Table 2:** Type of traumatic injuries

Traumatic injuries, N(%)	Fracture	Soft-tissue
Hand-wrist	15(10.9)	11(8.0)
Foot-ankle	16(11.6)	19(13.8)
Thigh-knee-leg	8(5.8)	14(10.1)
Upper-arm-elbow-forearm	4(2.9)	6(4.3)
Head-neck	3(2.2)	8(5.8)
Back	4(2.9)	5(3.6)
Shoulder	5(3.6)	2(1.4)
Thoracic-abdominal	2(1.4)^a^	4(2.9)
Other	0	12(8.7)^b^

^a^Rib fracture

^b^Soft-tissue contusions and lacerations occurred at surface area where the anatomical sites were hard to define

A clustering analysis of the anatomic traumatic injury positions revealed that of the 138 traumatic injuries in this study, 61 (44.2 %) had foot-ankle and hand–wrist injuries and 22 (15.9 %) had lower thigh-kneeleg injuries (Table 2). Fewer injuries were observed in the head-neck (11, 8.0 %), thoracic-abdominal region (6, 4.3 %) and back (9, 6.5 %). A total of 57 fracture cases (Fig. 1d) included 31 (54 %) with ankle–foot and hand–wrist fractures, 12 (21 %) with long bone limb fractures, 5 (9 %) with head-neck-chest fractures and 4 (7 %) with lower back involved fractures. No pelvis or hip fractures and severe head trauma or chest visceral injuries were observed.

As shown in Table 3, among non-traumatic patients, a total of 68 cases of respiratory and digestive system infections (approximately 73.9 %) were observed. These illnesses occurred significantly more frequently than other conditions, which included five of respiratory system infection induced by plateau pulmonary oedema and two of plateau brain oedema and a total of 19 (20.7 %) of dermatitis.

**Table 3:** Type of non-traumatic injuries

Non-traumatic injuries	N(%)
Infection	
Muscle-skeleton system	5(5.4)^a^
Respiratory system	38(41.3)
Digestive system	23(25)
Skin	19(20.7)
High altitude pulmonary oedema (HAPE)	5(5.4)^b^
High altitude cerebral edema (HACE)	2(2.2)^b^

^a^Muscle strain

^b^Induced by respiratory infection

### Personal protection equipments and injury sites

Patients were grouped into three relief working groups according to their personal protective equipment (PPE) as described in material and methods. The “military” relief working group was equipped with camouflage uniform and cap, combat boots and thin thread woven gloves. The “professional” relief working group was equipped with professional uniform and helmet, long boots, protective gloves with thick layers and mask. The “other” working group was not equipped with any particular equipment. A Pearson chi-square test revealed significant difference (*p* = 0.037, Pearson coefficient 0.314, *phi* coefficient 0.330, *Cramer’s* V coefficient 0.234) in injury incidence in different working groups according to their PPE (Table 4).

**Table 4:** Traumatic injury sites in different relief working groups^a^

N(%)	Relief working groups		
	Military	Professional	Other
Hand-wrist	16 (24.6)	2 (9.5)	8 (15.4)
Foot-ankle	11 (16.9)	5 (23.8)	29 (36.5)
Thigh-knee-leg	10 (15.4)	5 (23.8)	7 (13.4)
Upper-arm-elbow-forearm	6 (9.2)	1 (4.8)	3 (5.8)
Others	22 (33.8)	8 (38.1)	15 (28.8)
Total sites	65 (100)	21 (100)	52 (100)

^a^ *p* = 0.037, Pearson coefficient 0.314, *phi* coefficient 0.330, *Cramer’s* V coefficient 0.234

A further look at traumatic injuries, hand-wrist injuries constitute the major part of injuries that “military” relief working group endured accounting for 24.6 % of the total group injury sites while it only accounted for 9.5 % of the total injury sites in “professional” relief working group (Table 4). Given the fact that “military” was equipped with thin thread woven gloves and “professional” was equipped with protective gloves with thick layers, the difference in injury site distribution in different relief working groups indicates the thin thread woven gloves were not enough to protect soldiers from their relief/rescue work load and protective gloves with thick layers should be recommended for this group.

Likewise, combat boots and long boots both appeared effective in protecting rescuer from foot-ankle injuries. Foot-ankle injuries constitute 36.5 % of the total injury sites in the group with no personal protection equipments, while it accounted for 16.9 % in combat boots group and 23.8 % in long boots group (Table 4).

### Causes of injuries

**Fig. 2:**
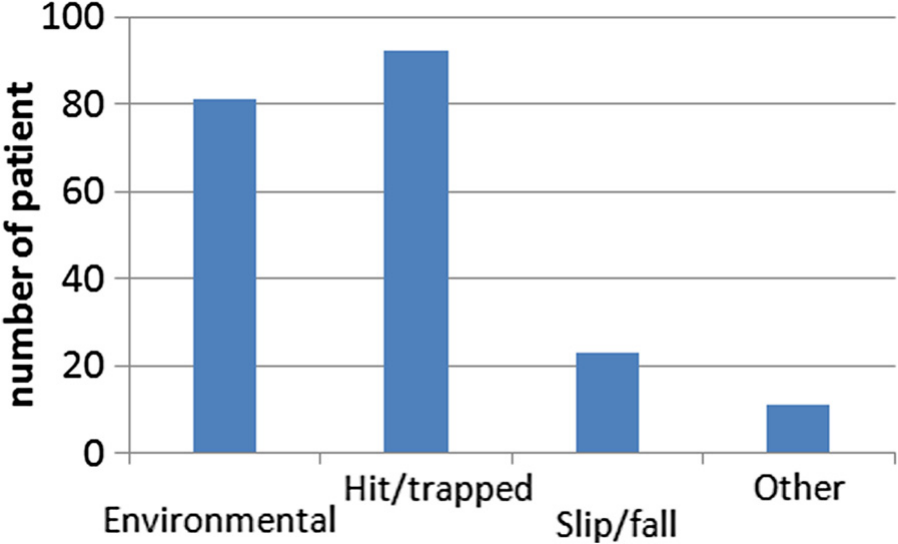
Causes of injuries in 207 rescue workers

The etiological analysis found that hitting/trapping by objects due to aftershocks and rescue was the leading cause of injuries, accounting for approximately 44.4 % of the total cases (92 cases, Fig. 2,). Environmental exposures including climate related factors and dietary factors was the second leading cause of injuries, accounting for 39.1 % of a total of 81 cases. Finally, 23 cases (11.1 %) were caused by slipping/falling related events during the rescue process.

## Discussion

The present study showed that the most frequent injury sites for professional rescue workers during the earthquake rescue work in high-altitude were the ankle-foot and hand-wrist. The results suggested that the specific high-altitude environment increased the challenges associated with earthquake relief.

Unlike previous studies of earthquake-related injuries [9–13], the present study was focused on injuries of relief workers only, in the Shigatse region of the Tibetan plateau following the ‘4.25’ Nepal earthquake disaster. Injuries happening in the principal earthquake were excluded since it was not relevant to rescue-work-incurred injuries. Earthquake relief activities were conducted under careful organization by the army and government, and the procedures used by disaster relief workers to implement disaster relief activities were planned with careful detail, that’s why no lethal injuries was observed in our study. However, our results indicated there was space for improvement regarding personal protection equipments for rescue workers. For example, boots and protective gloves with thick layers should be recommended to every rescue workers to reduce traumatic injuries on hands and feet.

Patients aged 20–39 years accounted for approximately 64.3 % of the total patients and most of whom were male. This was consistent with the fact that army comprised majority of earthquake relief rescuers. The army mainly comprises male soldiers and officers aged 16–35 years, resulting in a relatively lower proportion of female patients in our study. Children, women and the elderly was rare in this rescuer patient pool because they were moved to safe zones immediately after the earthquake and were not allowed to participate in most of the disaster relief efforts [14, 15].

The main cause of injury in this study was trauma resulting from hit and/or trapped by collapsed buildings following strong aftershocks and the secondary collapse of ruins caused by the principle earthquake. During the rescue process, relief workers must often dig deep into the ruins to rescue life. Unstable ruins would cause facet joint damage to the hand-wrist and foot-ankle [16, 17]. In this study, cumulative fractures of the hand-wrist and foot-ankle accounted for more than half of the total fractures, especially in military working group indicating insufficient PPE for the work load. Another reason might be that military are likely to be stronger, younger and better trained than herdsmen. Rescue workers are more likely to be injured in the plateau environment, as they will become fatigued and slow, experience power losses and encounter response delays to crises in a low-oxygen environment [18, 19].

In contrast to previous reports regarding the injuries incurred in ordinary people during principle earthquakes [20, 21], more single site injuries were observed while facet joint limb damage was found to be more common than chest-abdomen injury. This may be associated with the specific relief operation. Specifically, because most of the relief workers were well trained professionals and the rescue implementation was well planned and organized [16] to minimize personal injuries. To mitigate the influence of the plateau environment, the rescue workers were grouped into rescue echelons. Accordingly, frontline rescue workers could be replaced in a timely manner to avoid secondary injury caused by work overload.

Forty percent of the cases were found in this study with non-traumatic illness mainly due to environmental exposure including severe weather and dietary factors. Relief workers stayed in tents during the rescue period and were exposed to cold, wet weather conditions in the area with elevation of more than 4000 m (high altitude); in addition, many relief workers had no living experience in high altitudes (elevation >3500 m). Accordingly, the environmental conditions led to different types of altitude sickness and various other types of illness [22, 23], particularly respiratory infections, which accounted for 45 cases (21.7 %) as well as varying degrees of pulmonary oedema and cerebral oedema complications. The earthquake caused widespread landsides, leading to traffic blockades and lack of proper relief supply, particularly with regard to food and medical supplies. Gastrointestinal diseases caused by an improper diet accounted for approximately 10.0 % of all cases, and dermatitis accounted for approximately 8.3 % of the cases. The limitations of this study include the rather small size of subjects because the data were collected in one earthquake in a very short time.

In summary, the present study showed the distribution of injury sites for professional rescue workers during the earthquake rescue work, which might be useful for prevention of injuries in the future rescue work.

## Conclusion

The specific plateau environment and climate increased the burden and challenge in earthquake relief. Relief workers are more prone to limb and joint damage, single site injuries. The specific weather and dietary situation increased the incidence of respiratory and digestive diseases. The injury distribution data shown in this study demonstrated that effective organization and personnel protection can reduce the injury occurrences. The key is to strengthen the protection of vulnerable parts of the body. For personnel who are often involved in rescue work, such as soldiers and professional rescue team should receive the training and education of protection for reducing injuries. In the case of the poor environment in the plateau, more attention should be paid to the changes of the climate and food. This study could facilitate the organization and implementation of earthquake relief efforts in plateau areas, whilst providing counsel to related issues of injury and plateau disease prevention and clinical management in earthquake rescue.
